# Challenges in the Management of Upper Lid Keloid

**DOI:** 10.1155/2022/3032246

**Published:** 2022-03-25

**Authors:** Ruchi Goel, Samreen Khanam, Shalin Shah, Ravindra Kumar Saran

**Affiliations:** ^1^Ophthalmology, Maulana Azad Medical College, New Delhi, India; ^2^Pathology, Govind Ballabh Pant Hospital, New Delhi, India

## Abstract

A middle-aged lady presented with a firm, nontender mass on the left upper lid and area behind the left ear following lid reconstruction with postauricular graft for cicatricial ectropion 11 months prior. She had a similar mass on the right shin. She was diagnosed as a case of multiple keloids. Intralesional injection of triamcinolone acetonide suspension and 5-Fluorouracil (5-FU) in the upper lid keloid resulted in ulceration of its surface. Surgical excision, injection of 5-FU in the keloid bed with temporal forehead flap reconstruction, was performed. Occurrence of inadvertent postoperative wound infection with *Acinetobacter baumannii* was treated with local dressing with colistimethate sodium. Adjuvant therapy with topical imiquimod cream 5% was given subsequently for 24 weeks with no recurrence of the lid keloid after 16 months. The patient was managed using a combination of conservative and surgical therapy and multidisciplinary team work and kept on a long term follow-up.

## 1. Introduction

Wound healing is a natural dynamic process involving a complex interplay between regulatory mediators and involves stages of inflammation, proliferation, and remodeling. The phase of remodeling, which usually begins 3 weeks after tissue injury and continues for months thereafter, is primarily responsible for variation in scar quality. Abnormal regulation of the healing process can result in pathological scarring in the form of keloids and hypertrophic scars [1]. Keloids as compared to hypertrophic scars, appear slowly over months, extend beyond the wound causing greater aesthetic disfiguration and discomfort [2].

The occurrence of keloids has strong racial and genetic influences, with higher incidence seen in individuals with African, Asian, and Native American ethnicity (ranging from 5% to 16%) [3]. Familial cases have also been observed, and a number of genes and genetic loci identified, although no one specific gene has been found to be associated. Melanocytes appear to play a contributory role in keloid development as its incidence is very low in Caucasians (approximately, 0.1%), and has rarely been reported in individuals with albinism [4].

Keloids are mostly seen in wounds experiencing high tensile forces. In the head and neck region, the common sites for keloid formation are ear lobe, postauricular area, occipital scalp, and upper neck [5].

Involvement of upper eyelids is rare, possibly due to its inherent characteristics of thinness and laxity of skin. Many of the conventional treatment protocols may not apply to this region. Herein, we describe the challenges faced during the management of the upper lid keloid.

## 2. Case Presentation

A middle-aged lady presented with the chief complaint of a progressively increasing mass on her left upper eyelid and behind the left ear for the past 7 months which caused discomfort in wearing spectacles. She had a history of road traffic accident 11 months back during which she sustained injury to both lids of the left eye and fracture of the right leg bones. Her records revealed left upper lid reconstruction with postauricular graft 10 months prior for upper lid cicatricial ectropion with lagophthalmos. Two months following surgery, she started having itching, redness, and thickening of the left upper lid and postauricular region.

**Figure 1:**
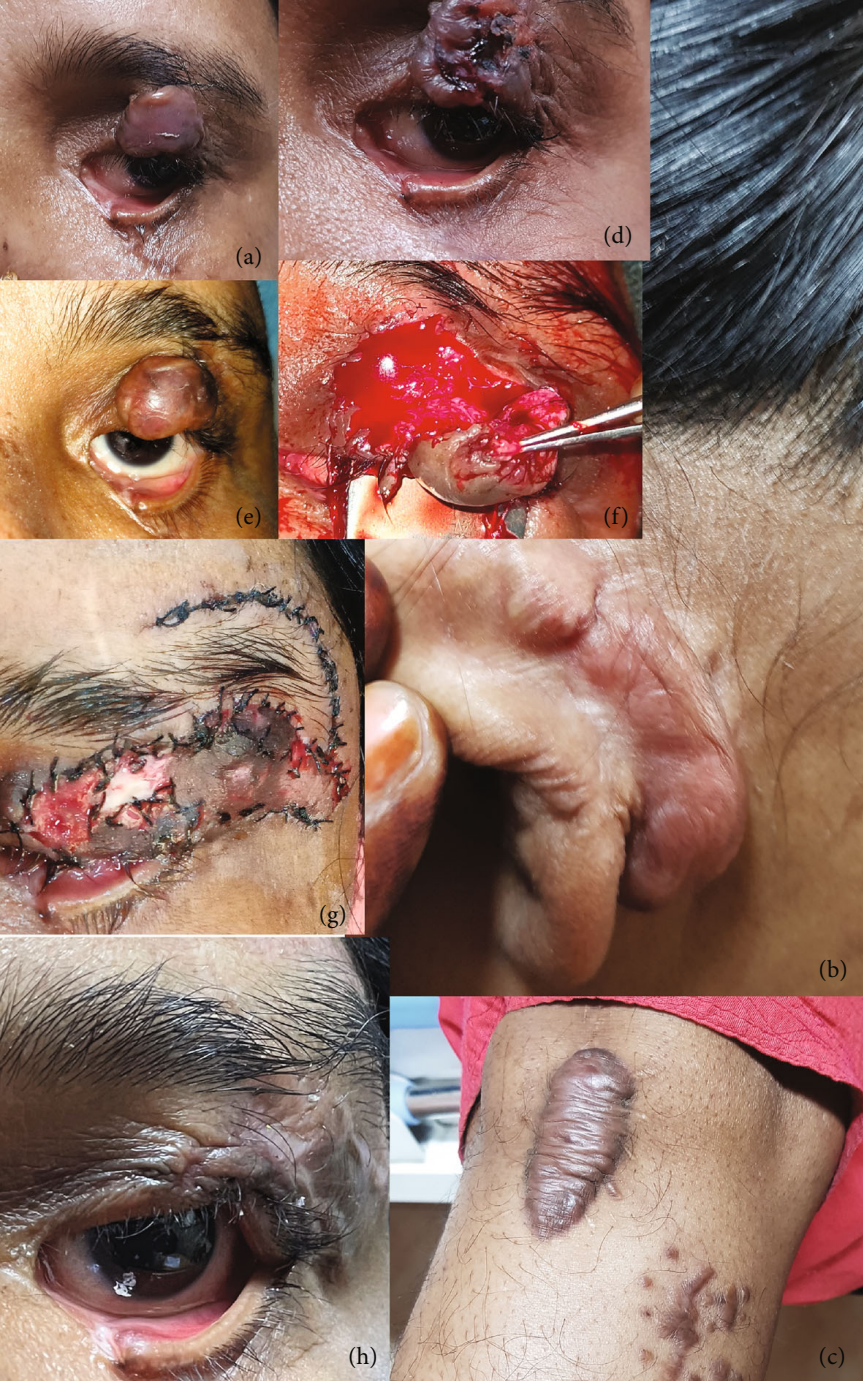
(a) A well-defined mass seen on the left upper lid (1.5 cm × 1.2 cm × 1 cm) involving the lid margin. (b) Thickened mass seen in the postauricular area. (c) Hyperpigmented lesions seen on the right leg. (d) Ulceration of keloid surface following intralesional 5-FU (50 mg/mL) and crystalline TAC (40 mg/mL) injection. (e) Regrowth of keloid after discontinuation of 5-FU (50 mg/mL) and crystalline TAC (40 mg/mL) injection. (f) Excision of keloid. (g) Development of postsurgical wound infection. (h) Clinical appearance after 16 months of upper lid keloid excision.

Upon presentation to our clinic, her best corrected visual acuity was 20/20 in both eyes. A thickened mass (1.5 cm × 1.2 cm × 1 cm) involving the lid margin was seen on the left upper lid (Figure 1(a)). It was nontender and firm in consistency with no overlying skin changes. There was limitation in the left eyelid excursion. The left lower eyelid had 1/3^rd^ medial defect with canalicular injury and cicatricial ectropion. Syringing was patent through the upper punctum. Bell's phenomenon was good. The extraocular adenexa of the right eye as well as the anterior and posterior segments of both the eyes were unremarkable.

Firm lesions (4 cm × 5 cm × 5 cm) were seen in the left postauricular region and anterior aspect of the right leg (4 cm × 1.5 cm × 0.5 cm) and right medial leg (Figures 1(b) and 1(c)). There was no history of previous injury or ear piercing. Her father gave history of similar lesions on his chest wound 20 years back for which he had undergone excision with no further recurrence.

Her blood group was A positive. Haemogram, blood sugar, and kidney function tests were within normal limit.

## 3. Treatment

**Figure 2:**
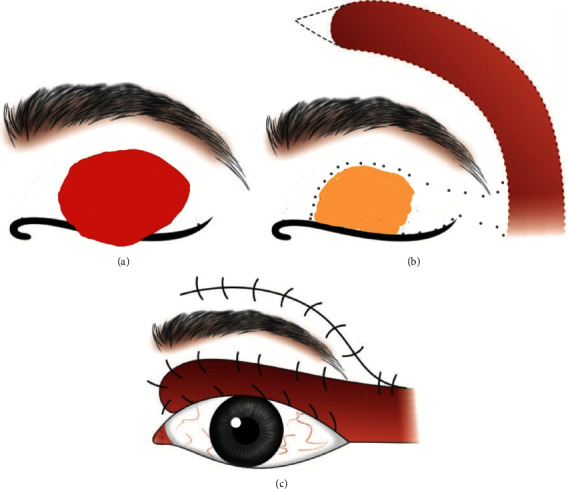
(a) A well-defined mass seen on the left upper lid involving the lid margin. (b) After excision of the mass, the skin was undermined to decrease the size of the anterior lamellar defect and a lateral forehead rotational flap was raised. (c) The lateral forehead flap was mobilized to cover the defect, and the wound was closed without tension on suture line.

A diagnosis of multiple keloids was made, and treatment was commenced for the upper lid lesion. She was advised to apply silicone gel (Scarend silicone gel, Mankind pharmaceuticals) twice a day for a month. Three doses of intralesional 5-Fluorouracil (5-FU) (50 mg/mL) and crystalline triamcinolone acetonide suspension (TAC) (40 mg/mL) in 3 : 1 ratio were then given at 3 weekly intervals. There was a shrinkage in the size of the lesion (1.3 cm × 1.2 cm × 0.5 cm), but further injections were stopped following development of ulceration on the keloid surface (Figure 1(d)). The ulcer healed in a month; however, the patient declined further injections. She returned 8 months later with increase in the size of the upper lid lesion (1.6 cm × 1.3 cm × 1.2 cm) (Figure 1(e)). Complete surgical excision of the eyelid keloid was performed, and the defect was closed with a lateral forehead rotational flap under asepsis (Figure 1(f), Figure 2). One millilitre of 5-FU (50 mg/mL) was injected at the wound site intraoperatively. She was discharged on tablet Augmentin 625 mg 8 hourly, tablet Ibuprofen 400 mg 8 hourly, and local application of tobramycin eye ointment twice daily.

**Figure 3:**
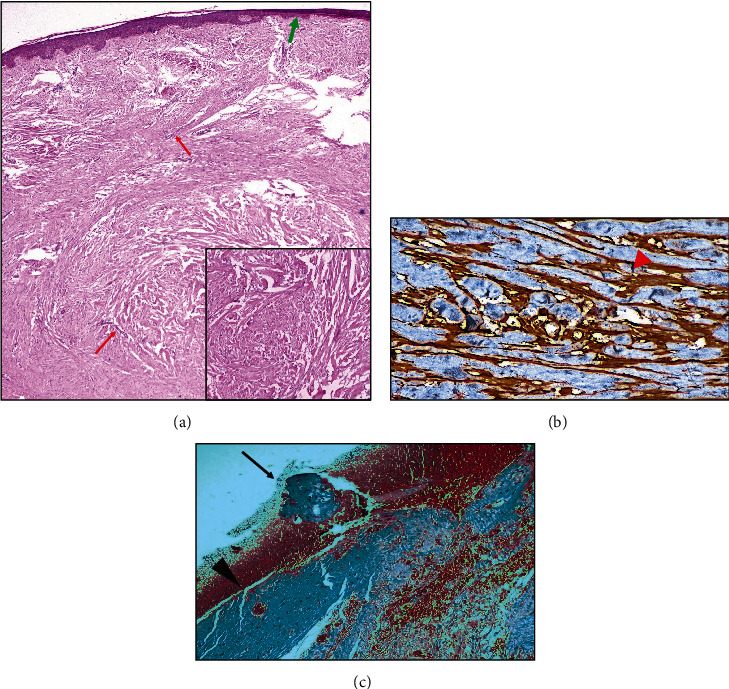
(a) Histopathology showing stretched out epithelium (green arrow), paucicellular thick collagen bundles, and few widely scattered blood vessels (red arrows) (hematoxylin-eosin stain-HE, 4x). Inset shows disorganised, large, hyalinised collagen fibres (HE, 20x). (b) TGF-*β* marker shows focal positivity (red arrowhead) in the interstitium suggesting active fibroblast activity (Immunohistochemistry, 40x). (c) Thick collagen bundles (black arrowhead) with a nodule of thick collagen fibres (black arrow) at the margin of the lesion (Masson-trichome stain, 10x).

Histopathological features of the excised lesion were suggestive of keloid. The interstitium showed focal positivity on transforming growth factor beta (TGF-*β*) marker. There were mature collagen fibres and absence of myofibroblasts in the marginal area (Figure 3).

In the follow-up visit on the 7^th^ postoperative day, tissue discoloration and a pus point were noticed adjacent to the lid suture (Figure 1(g)). The discharge was sent for microbiological examination and *Acinetobacter baumanii,* sensitive to colistin, and tigecycline *was* isolated. Daily dressing with application of colistimethate sodium (CMS) (4.5 million IU) (Xylistin, Cipla) colistin powder was done for 14 days, which resulted in satisfactory wound healing. The patient was advised local application daily of imiquimod 5% cream for 24 weeks.

## 4. Outcome and Follow-Up

At 16 months of follow up, there was no evidence of recurrence of keloid on the left upper lid (Figure 1(h)). She was advised to keep a watch for symptoms suggestive of keloid recurrence such as firmness and redness of the scar or surrounding area and itching.

Her postauricular keloid was excised by the plastic surgery department. She was not inclined for treatment of her leg lesions or left lower lid ectropion.

## 5. Discussion

The development of keloid results in cosmetic disfigurement, pruritus, pain, and hyperesthesia resulting in impairment of health-related quality of life. It is usually seen in individuals between 10 and 30 years but can affect beyond sixth decade [6, 7]. Lesser frequency of scar formation in elderly is attributed to reduction in skin tension and sebaceous gland activity. Associated risk factors include dark skin pigmentation, positive family history, blood group A, and hormonal changes occurring during puberty or pregnancy [8].^,^ Our patient was a quadragenarian, with brown skin colour, a positive family history, and blood group A.

Keloids most commonly affect upper arms, skin overlying joints, chest, shoulders, and ear lobes. High skin tension plays a critical role in scar formation. Tension across an incision tends to pull the skin edges apart and the wound, in response increases the collagen deposition. Areas with rich blood supply like face are known to heal with finer scars [1]. In our patient, possibly, in postinjury, there was a development of a large upper lid defect which led to extensive cicatrization. Closure of both donor and recipient site under tension during the reconstruction procedure, in a predisposed patient, could have led to keloid formation in both the donor and recipient area.

In the early stages of keloid maturation, corticosteroids, 5-Fluorouracil (5-FU), bleomycin, and verapamil have been used for treatment [9]. Application of silicone gel or hydrocolloid dressings prior to intralesional injection of 5FU or TAC results in scar softening [10]. Surgical excision is indicated in nonreactive refractory keloids and often combined with adjuvant therapy, such as corticosteroid, 5-FU, radiotherapy, 5% imiquimod application, or cryotherapy to lower the recurrence rates [11, 12].

TAC is the most widely used corticosteroid with reported recurrence rates varying from 33% to 50% when used alone [3]. 5-FU, a pyrimidine analog, inhibits the proliferation and differentiation of myofibroblasts. It is used as monotherapy or in combination with steroids. The reported side effects of intralesional steroid are pain, skin atrophy, alteration in skin pigmentation, and formation of telangiectasias. The 5-FU may cause local erythema, pain, hyperpigmentation, ulceration, a burning sensation, and sloughing [11, 13]. We used a combination therapy of 5-FU and TAC; however, following three doses of intralesional injection, though the volume decreased, an ulcer developed on the surface of keloid. Later, following discontinuation of injection, the keloid again increased in size.

Wide surgical excision of keloid is recommended to prevent regrowth [14]. Wide excision was not suitable for the upper lid keloid, as in our case. The clinically visible mass was thus excised, 5-FU was injected in the wound bed, and the wound meticulously closed after mobilization of temporal forehead flap to avoid skin tension. Intramarginal core excisions have low recurrence rates in mature keloids [15]. In general, recurrence rates of 0 to 44% have been reported with surgical excision without adjuvant therapy [2]. The plausible options after excision in our case included local injection of steroids, 5 FU, and interferon alpha-2b. Also, topical imiquimod cream 5% and application of an absorbent material soaked in 1 mg/mL of mitomycin C could have been used. Compressive therapy successfully used in ear keloids was not appropriate for upper lid. Radiation therapy, an effective adjunctive therapy after excision, is usually reserved for refractory keloids [5]. Since our patient was unwilling for injectables, topical imiquimod cream 5% (Imiquad, Mecoson labs private limited, India) was prescribed twice a day for 24 weeks after control of postoperative wound infection. The patient did not show recurrence of upper lid keloid till the last follow-up visit.

Despite detailed instructions to maintain wound hygiene, the patient developed postoperative infection with *Acinetobacter baumanii,* sensitive to colistin and tigecycline. Local application of colistin instead of systemic administration, in susceptible infections, prevents renal toxicity and reduces the treatment cost [16]. In our case also, the infection was successfully controlled with local application of colistimethate sodium; however, the wound healed with scarring of lid margin.

To conclude, keloid of the eyelid, a rarely reported clinical entity, can be managed by a combination of surgical and nonsurgical treatment modalities. Given the occurrence of keloid at multiple sites and unpredictable duration of recurrence, a multidisciplinary coordination and a long-term follow-up is required.

## Data Availability

The investigations to support the findings are included in the text.
